# The association between school climate and mathematics achievement among middle school students: the serial indirect roles of mathematical bullying and psychological resilience and the moderating roles of grade level and gender

**DOI:** 10.3389/fpsyg.2026.1968781

**Published:** 2026-10-05

**Authors:** Baris Demir, Rumeysa Beyazhancer

**Affiliations:** 1 Kocaeli University, Izmit, Türkiye; 2 Department of Mathematics Education, Faculty of Education, Bursa Uludag University, Bursa, Türkiye

**Keywords:** mathematical bullying, mathematics achievement, moderation, psychological resilience, school climate, serial mediation

## Abstract

**Introduction:**

School climate and bullying have both been associated with students' mathematics achievement. However, the roles of mathematics-specific bullying and psychological resilience in this association remain insufficiently understood. This cross-sectional study examined the serial indirect roles of mathematical bullying and psychological resilience in the association between school climate and mathematics achievement. It also evaluated the moderating roles of grade level and gender in specific associations within the model.

**Methods:**

Participants were 708 students attending three public middle schools. School climate, mathematical bullying, and psychological resilience were assessed using the Brief School Climate Scale, Mathematical Bullying Victimization Scale, and Brief Resilience Scale, respectively; mathematics achievement was determined from students' course grades. Confirmatory factor analysis, Pearson correlation analysis, multiple regression, serial indirect-association analysis, and moderation analysis were conducted using IBM SPSS Statistics, version 29.0 (IBM Corp., Armonk, NY, USA); IBM SPSS Amos, version 29.0 (IBM Corp., Armonk, NY, USA); and PROCESS macro, version 4.1 (Andrew F. Hayes, Calgary, AB, Canada).

**Results:**

School climate was negatively associated with mathematical bullying and positively associated with psychological resilience and mathematics achievement. Mathematical bullying was negatively associated with both psychological resilience and mathematics achievement, whereas psychological resilience was positively associated with mathematics achievement. The total association between school climate and mathematics achievement was positive and statistically significant (β= 0.207, *p* < 0.001). The indirect association via mathematical bullying was not statistically significant (β= 0.032, *p* < 0.281; 15.6%). In contrast, the indirect association through psychological resilience (β= 0.025, *p* < 0.039; 11.9%) and the serial indirect association through mathematical bullying and psychological resilience (β= 0.016, *p* < 0.024; 7.7%) were significant. The total indirect association was significant (β= 0.073, *p* < 0.024) and accounted for 35.2% of the total association. The negative association between mathematical bullying and psychological resilience was stronger among sixth-grade students and among girls. The positive association between school climate and mathematics achievement was significant only among seventh-grade students and among boys, whereas the negative association between mathematical bullying and mathematics achievement was significant only among boys.

**Discussion:**

The findings highlight the relevance of psychological resilience and of the serial indirect pathway, through mathematical bullying and psychological resilience, in the association between school climate and mathematics achievement. Given the cross-sectional design, the results should be interpreted as theoretically specified statistical associations, rather than as evidence of temporal or causal mechanisms.

## Introduction

Differences in mathematics achievement cannot be accounted for solely by students‘ cognitive competencies. Students' mathematics-related self-beliefs, motivation, and attitudes are also associated with mathematics achievement (Lee and Stankov, 2018; Pitsia et al., 2017). These individual characteristics are not independent of the social and instructional context in which learning occurs. Evidence from different research designs indicates that school climate characteristics such as teacher support, student relationships, safety, belonging, and order are associated with student engagement, motivation, and academic achievement (Berkowitz et al., 2017; Konold et al., 2018; Daily et al., 2019; Fan and Williams, 2018; Maxwell et al., 2017; Lee et al., 2026). Research syntheses likewise show that positive school and classroom climates are associated with higher academic achievement (Demirtaş-Zorbaz et al., 2021; Erdem and Kaya, 2024).

The association between school climate and academic outcomes is not limited to conditions that support learning. School climate characteristics such as teacher and peer support, clear behavioral rules, safety, and adult intervention in bullying have been associated with lower levels of bullying and victimization (Gage et al., 2014; Cornell et al., 2015; Låftman et al., 2017; Mucherah et al., 2018; Acosta et al., 2019; Marchante et al., 2022). Meta-analytic findings showing a negative association between positive school climate and bullying victimization further support this evidence (Li et al., 2025). These studies suggest that bullying should be understood not merely as a behavior arising from students' individual characteristics, but rather as an experience associated with the school's social and relational conditions.

A negative association between bullying victimization and academic achievement has been documented across different samples and research methods (Nakamoto and Schwartz, 2010; Murphy et al., 2022; Özyildirim and Karadag, 2024). This association is also supported specifically for mathematics achievement by large-scale national and international data (Konishi et al., 2010; Román and Murillo, 2011; Ponzo, 2013; Delprato et al., 2017; Rutkowski and Rutkowski, 2018; Carrasco et al., 2022; Ren et al., 2025; Campillo González et al., 2025; Mokgwathi, 2026). This evidence indicates that the academic correlates of bullying are not confined to general achievement and warrant separate examination in the context of mathematics learning.

Nevertheless, evidence of an association between bullying and mathematics achievement does not by itself clarify the processes through which this association may operate. Previous research has identified psychological difficulties (Murphy et al., 2022), cognitive-motivational factors and academic engagement (Samara et al., 2021), and teacher–student relationships and school belonging (Carrasco et al., 2022; Ren et al., 2025) as potential mechanisms linking bullying with academic outcomes. However, the role of psychological resilience—the capacity to maintain or regain adaptation in the face of adverse experiences—in the pathway linking school climate with mathematics achievement remains insufficiently understood.

Although research on general bullying provides an important foundation, it may not fully capture the specific power relations found in mathematics lessons. In mathematics classrooms, correct answers, calculation speed, and achievement levels can be openly compared, and mathematical competence may become an indicator of social power among students. Küçükgençay (2025) conceptualized mathematical bullying as the transformation of differences in mathematical knowledge and achievement into systematic abuse of power in interpersonal relationships. In addition to ridicule based on mathematical performance, mathematical bullying encompasses interference with a student's materials, avoidance of participation because of fear of humiliation, and negative behaviors perceived from the teacher (Küçükgençay and Peker, 2024; Küçükgençay, 2025). The inclusion of perceived teacher-related behaviors within the instructional dimension of mathematical bullying makes mathematical bullying a construct specific to mathematics instruction, rather than one restricted to traditional forms of peer bullying.

Examining these variables together requires an approach that considers, within the same model, the student's school context and individual psychological resources. From the perspective of Bronfenbrenner (1979) Ecological Systems Theory, school climate can be positioned as a contextual characteristic and mathematical bullying as a domain-specific risk experience that may occur within that context. Within Masten's (2014) resilience framework, psychological resilience can be viewed as an individual resource that may contribute to maintaining adaptation in the face of adverse experiences. This framework provides a theoretical basis for bringing school climate, mathematical bullying, psychological resilience, and mathematics achievement together in a single explanatory model.

This study aimed to examine the separate and serial indirect associations between school climate and mathematics achievement through mathematical bullying and psychological resilience. It also examined whether the specified associations varied by grade level and gender. Accordingly, the study centers on domain-specific bullying experiences that arise in relation to mathematics performance and instruction and integrates the school's social context, the risk of mathematical bullying, students' psychological resources, and mathematics achievement into a single model. The following research question guided the study:

RQ. To what extent is the association between school climate and mathematics achievement statistically indirect through mathematical bullying and psychological resilience, separately and sequentially, and do specific associations within the model vary by grade level and gender?

## 2 Theoretical background and hypotheses

### 2.1 School climate and mathematics achievement

School climate is a multidimensional construct encompassing shared experiences and perceptions of the norms, values, interpersonal relationships, teaching and learning practices, and institutional arrangements that shape school life. Thapa et al. (2013) conceptualized school climate in terms of safety, relationships, teaching and learning, the institutional environment, and the school improvement process, whereas Wang and Degol (2016) classified it across the domains of academics, community, safety, and institutional environment. Both approaches indicate that school climate extends beyond physical conditions to include teacher and peer relationships, the implementation of school rules, social and emotional safety, and support for learning.

The positive association between school climate and academic achievement is supported by research syntheses, multilevel analyses, and studies conducted across different educational levels (Berkowitz et al., 2017; Konold et al., 2018; Daily et al., 2019; Demirtaş-Zorbaz et al., 2021; Erdem and Kaya, 2024). Berkowitz et al. (2017) found that positive school climate was associated with academic achievement and could provide a protective context for disadvantaged students. Konold et al. (2018) reported that schools‘ disciplinary structure and support for students were associated with student engagement, which in turn was associated with academic achievement. Daily et al. (2019) likewise identified significant associations between different dimensions of school climate and middle and high school students' self-reported academic achievement. These findings suggest that the association between school climate and achievement may be linked not only to order and safety but also to how students are supported and participate in learning processes.

Studies examining the association between school climate and mathematics achievement suggest that motivational, social, and contextual processes may be involved (Fan and Williams, 2018; Maxwell et al., 2017; Teng, 2020; Lee et al., 2026). Fan and Williams (2018) found that mathematics self-efficacy and intrinsic motivation mediated the association between students' perceptions of school climate and mathematics achievement. Maxwell et al. (2017) showed that school identification constituted a mediating mechanism in the association between school climate and numeracy achievement. Teng (2020) reported that positive student–teacher relationships, an orderly disciplinary climate, and positive student behavior were associated with smaller family-background-related disparities in mathematics achievement. The multilevel findings of Lee et al. (2026) further indicate that the links between student- and school-level characteristics related to school climate and mathematics achievement cannot be understood solely at the individual level.

Taken together, these studies indicate that the association between positive school climate and mathematics achievement may be linked to individual processes such as student engagement, mathematics self-efficacy, intrinsic motivation, and school identification, as well as to relational and disciplinary characteristics of the school. On the basis of this theoretical and empirical evidence, the following hypothesis was formulated:

H1. Students' perceptions of a positive school climate are positively associated with their mathematics achievement.

### 2.2 School climate, mathematical bullying victimization, and mathematics achievement

The association between school climate and bullying has been examined in relation to characteristics such as disciplinary structure, teacher support, peer relationships, safety, and competitiveness (Biernbaum and Lotyczewski, 2015; Cornell et al., 2015; Huang et al., 2024; Låftman et al., 2017; Montero-Carretero et al., 2021; Mucherah et al., 2018). Biernbaum and Lotyczewski (2015) found that the quality of interpersonal relationships, in particular, was associated with students' involvement in bullying. Cornell et al. (2015) showed that a strong disciplinary structure and student support were associated with lower levels of bullying and victimization. Låftman et al. (2017) identified associations between students' awareness of school rules, their perceptions that adults intervened in bullying, and lower victimization. Mucherah et al. (2018) likewise found that a positive school climate and teachers' actions to stop bullying were associated with fewer reports of bullying and victimization. These findings suggest that bullying is not merely an individual problem among students but is associated with the relational, supportive, and regulatory characteristics of the school. Consistent with this view, Wang et al. (2013) proposed that an unhealthy and unsupportive school climate, characterized by negative teacher–student relationships and attitudes that tolerate bullying, may provide a social context in which bullying can occur and persist.

Social and psychological mechanisms such as school connectedness, self-esteem, empathy, and teacher-student relationships may play a role in the association between school climate and bullying (Acosta et al., 2019; Zhao et al., 2021; Montero-Carretero et al., 2021; Marchante et al., 2022). Acosta et al. (2019) found that students who perceived a positive school climate reported less physical, emotional, and cyberbullying and that school and peer connectedness, assertiveness, and empathy contributed to accounting for these associations. Zhao et al. (2021) showed that self-esteem mediated the negative association between school climate and bullying victimization. Montero-Carretero et al. (2021) reported that different dimensions of school climate were associated with victimization, moral disengagement, and empathy. Marchante et al. (2022) found that, during the transition to middle school, changes in school climate characteristics such as teacher-student and student-student relationships, safety, fairness of rules, and liking school were associated with the development of bullying and victimization behaviors.

The competitive nature of school climate is also relevant to the conditions under which bullying occurs. Huang et al. (2024) found that a competitive school climate was positively associated with bullying victimization and that school belonging mediated this association. Although the vocational secondary education context of the study limits direct generalizability, the finding suggests that school environments that emphasize achievement and competition may be associated with power relations among students. This relationship is particularly relevant in mathematics classrooms because correct answers, calculation speed, and achievement levels can be compared visually among students.

Although general bullying research provides an important foundation, it may not fully account for negative interpersonal experiences arising from mathematical achievement and competence. Küçükgençay (2025) conceptualized mathematical bullying as the transformation of differences in mathematical knowledge and achievement into systematic abuse of power in interpersonal relationships. The Mathematical Bullying Victimization Scale comprises four dimensions—verbal, emotional, physical, and instructional-mathematical—and includes ridicule based on mathematics achievement, avoidance of participation due to anticipated humiliation, unauthorized interference with students' materials, and negative behaviors perceived as originating from teachers. This content gives the instrument a broader operational scope than conventional measures limited to peer bullying; the findings should therefore be interpreted with reference to the distinct dimensions and specific content of the scale.

In the present study, mathematical bullying is conceptually distinguished from general bullying by the mathematics-specific basis and context of the power relation: differences in mathematical knowledge, achievement, errors, or visible performance constitute the grounds on which disparagement, exclusion, interference, or other negative interpersonal behaviors occur (Küçükgençay and Peker, 2024; Küçükgençay, 2025). For the purposes of the present model, mathematics anxiety and fear of failure are not treated as equivalent to mathematical bullying because they do not, by themselves, denote an interpersonal experience of power or mistreatment. Likewise, avoidance of classroom participation is not interpreted as bullying itself, but as a response represented within the emotional content of the instrument. Teacher-related negative behaviors are also distinguished from conventional peer bullying in terms of their source and the structure of the power relationship. However, they remain within the operational scope of the present study because the scale includes them in its instructional-mathematical dimension (Küçükgençay, 2025). Accordingly, the findings refer specifically to the broad operationalization provided by the Mathematical Bullying Victimization Scale and should not be interpreted as encompassing all forms of mathematics anxiety, fear of failure, participation avoidance, or teacher mistreatment.

A negative association between bullying victimization and mathematics achievement has been documented across countries and large-scale datasets (Konishi et al., 2010; Delprato et al., 2017; Rutkowski and Rutkowski, 2018; Carrasco et al., 2022; Ren et al., 2025; Campillo González et al., 2025; Mokgwathi, 2026; TIMSS and PIRLS International Study Center, 2020). Küçükgençay (2025) also found that students in the low mathematics achievement group reported higher mathematical bullying victimization scores. However, this group comparison does not establish the direction of the association. Low mathematics achievement may be associated with a greater risk of victimization, and negative experiences related to mathematical performance may also be associated with students' classroom participation and academic achievement.

The association between school climate and mathematics achievement may involve multiple social and psychological mechanisms. Positive school climate is associated with lower bullying victimization, while lower victimization is associated with greater academic engagement, school belonging, and mathematics achievement (Acosta et al., 2019; Carrasco et al., 2022; Li et al., 2025; Ren et al., 2025). However, most existing studies have focused on general peer bullying; the role of mathematical bullying, which arises in the context of mathematical performance and instructional processes, in the association between school climate and mathematics achievement has not been directly examined. Accordingly, a positive school climate was expected to be associated with lower mathematics-related bullying victimization, which, in turn, was expected to be associated with higher mathematics achievement.

H2. There is a statistically significant indirect association between school climate and mathematics achievement through mathematical bullying victimization.

### 2.3 School climate, psychological resilience, and mathematics achievement

Psychological resilience refers to the capacity to maintain or regain adaptation in the face of difficulties, stressors, and adverse experiences (Masten, 2014). Mathematical resilience is a domain-specific construct that has been conceptualized both as successful mathematical performance despite disadvantage and as part of students' mathematical identity, including their persistence and adaptive responses to difficulties in learning mathematics (Kooken et al., 2016; Xenofontos and Mouroutsou, 2023). Academic resilience, by contrast, concerns adaptation in the face of serious and persistent adversity in education (Cassidy, 2016). Although related, these concepts do not represent the same construct. In this study, psychological resilience is treated not as a characteristic specific to mathematics lessons or to academic difficulties, but as a general individual resource that may help students maintain psychological functioning when facing risks such as mathematical bullying.

A positive association between school climate and psychological resilience is supported by studies conducted across different educational levels and student outcomes (Aldridge et al., 2020; Luo et al., 2023; Fang et al., 2025; Huang et al., 2025; Sahib et al., 2025; Yan and Li, 2025). Aldridge et al. (2020) found that different dimensions of school climate significantly accounted for variation in students‘ psychological resilience. Luo et al. (2023) reported that positive school climate was positively associated with perceived social support and psychological resilience, which independently and sequentially mediated the association between school climate and prosocial behavior. Huang et al. (2025) likewise found that psychological resilience mediated the association between positive school climate and adolescents' negative emotions. The longitudinal findings of Sahib et al. (2025) further indicated that a positive school climate was associated with greater subsequent psychological resilience both directly and indirectly through school identification.

The association between school climate and psychological resilience has also been examined in higher education samples (Fang et al., 2025; Yan and Li, 2025). Fang et al. (2025) found that psychological resilience mediated the association between school climate and socio-emotional competence. Yan and Li (2025) identified school climate as a significant statistical predictor of psychological resilience. However, because these studies were conducted with university students, their findings should be treated as complementary evidence when considering middle school students.

Direct evidence on the association between psychological resilience and mathematics achievement remains limited. Demir (2023) reported a positive association between psychological resilience and mathematics achievement among middle school students. Other findings indicate that the association between psychological resilience and general academic achievement may not always be direct. García-Martínez et al. (2022) found no direct association between psychological resilience and academic achievement among university students but reported an indirect association through self-concept. This finding suggests that additional psychological and academic mechanisms may underlie the association between psychological resilience and academic outcomes.

Consistent with the theoretical positioning outlined above, positive school climate was expected to be associated with psychological resilience, with students‘ perceptions of safety, support, and belonging providing a theoretical rationale for this association. Psychological resilience, in turn, was expected to be associated with students' continued effort and engagement in the face of social and academic difficulties in learning mathematics. School climate was therefore expected to have an indirect association with mathematics achievement through psychological resilience.

H3. There is a statistically significant indirect association between school climate and mathematics achievement through psychological resilience.

### 2.4 Mathematical bullying victimization and psychological resilience

The negative association between bullying victimization and psychological resilience, as well as the role of resilience in the psychological correlates of bullying, has been examined in several studies (Ime, 2024; Anderson et al., 2022; Gkatsa and Rachioti, 2026). The systematic review and meta-analysis by Moore et al. (2017) showed that bullying victimization in childhood and adolescence was associated with various psychosocial problems. Ime (2024) identified bullying victimization and loneliness as negative statistical predictors of psychological resilience. Gkatsa and Rachioti (2026) also found a negative association between bullying victimization and psychological resilience. Anderson et al. (2022) reported that bullying experiences were associated with lower psychological resilience and higher symptoms of depression and anxiety and that psychological resilience partially mediated these associations. These findings suggest that bullying victimization is associated not only with students' emotional adjustment but also with the psychological resources they draw on to cope with difficulties.

In bullying research, psychological resilience has been considered both a psychological resource associated with bullying experiences and a mechanism that may buffer their negative correlates (Lin et al., 2022; Shemesh and Heiman, 2021; Zhang et al., 2024). Lin et al. (2022) found that psychological resilience could serve a protective role in the associations between different types of bullying victimization and depression. Shemesh and Heiman (2021) reported that psychological resilience and self-concept statistically mediated the association between bullying victimization and adolescents' sense of wellbeing. Zhang et al. (2024) found that psychological resilience moderated the associations between bullying victimization and wellbeing in different directions: it attenuated some associations involving physical victimization but strengthened certain associations involving verbal and relational victimization. These findings suggest that the role of psychological resilience may vary according to the form of victimization and the wellbeing outcome examined. More broadly, observational and cross-sectional evidence does not establish that bullying victimization reduces psychological resilience.

Initial findings on the associations between mathematical bullying and students' emotional and motivational characteristics indicate that mathematical bullying is negatively associated with mathematics self-efficacy and attitudes toward mathematics and positively associated with mathematics anxiety Küçükgençay, (2025). The same study found that attitudes toward mathematics mediated the association between mathematical bullying and mathematics anxiety and that the sequential indirect association through self-efficacy and attitudes was significant. Complementing this construct-specific evidence, Kaplan (2026) found across six countries that general bullying victimization was consistently associated with higher mathematics anxiety and, in most countries, with lower perceived control in mathematics. Evidence from general bullying research also suggests that the association between bullying victimization and academic achievement may involve serial school-related processes. Li et al. (2020) found that school belonging, and academic engagement serially mediated the association between bullying victimization and academic achievement, although the pattern differed by gender. However, the associations of ridicule based on mathematical performance and of perceived negative teacher behaviors with students' general psychological resilience have not been examined directly. This gap suggests the value of examining how mathematical bullying is associated with students' psychological resources.

Although empirical evidence separately supports the associations between (1) school climate and mathematical bullying, (2) bullying and psychological resilience, and (3) psychological resilience and mathematics achievement, no study has examined these variables together within a single model of serial indirect associations. Examining the serial indirect associations through mathematical bullying and psychological resilience in the association between school climate and mathematics achievement, therefore, represents one of the study's original contributions.

H4. There is a statistically significant serial indirect association between school climate and mathematics achievement sequentially through mathematical bullying victimization and psychological resilience.

### 2.5 Grade-level and gender differences

Studies indicate that students' forms of involvement in bullying, their experiences of mathematical bullying, and the association between bullying and mathematics achievement may vary by gender (Obregón-Cuesta et al., 2022; Küçükgençay and Peker, 2024; Küçükgençay, 2025; Gimenez et al., 2024; Ren et al., 2025). Obregón-Cuesta et al. (2022) found that bullying roles differed by gender and that boys were more highly represented in the bully/victim group. Research on mathematical bullying, however, found that girls had higher total and emotional victimization scores than boys (Küçükgençay and Peker, 2024; Küçükgençay, 2025). Ren et al. (2025) reported that the negative association between bullying and mathematics achievement was stronger among girls, whereas Gimenez et al. (2024) found a more pronounced disadvantage in mathematics achievement among boys exposed to bullying. These differences suggest that the role of gender may vary across countries, samples, and educational contexts, and should be examined not only by mean scores but also by the strength of associations in the model.

Studies also indicate that bullying patterns and the association between bullying and academic outcomes may vary by grade level (Obregón-Cuesta et al., 2022; Özyildirim and Karadag, 2024; Küçükgençay and Peker, 2024; Küçükgençay, 2025). The meta-analysis by Özyildirim and Karadag (2024) found that the academic achievement difference between bullied and non-bullied students was larger in Grade 4 than in Grade 8. Subject domain also moderated this association, with a larger difference in mathematics achievement than in science achievement. Küçükgençay and Peker (2024) found no significant grade-level difference in total mathematical bullying scores. Similarly, Küçükgençay (2025) found no grade-level difference in the total score, although Grade 8 students scored higher than Grade 7 students on the instructional bullying dimension. These findings suggest that grade-level differences may emerge not in mathematical bullying as a whole, but rather in specific dimensions or in the association between mathematical bullying and academic outcomes.

The lack of a consistent pattern in findings concerning gender and grade level indicates that these variables should be examined not only as controls but also as potential moderators of specific associations in the model. Accordingly, the study tested whether grade level moderated the associations between (a) mathematical bullying and psychological resilience, and (b) school climate and mathematics achievement, and whether gender moderated the associations between (a) mathematical bullying and psychological resilience, (b) school climate and mathematics achievement, and (c) mathematical bullying and mathematics achievement.

H5. Grade level and gender moderate specific associations within the research model.

H5a. Grade level moderates the associations between mathematical bullying and psychological resilience, and between school climate and mathematics achievement.

H5b. Gender moderates the associations between (a) mathematical bullying and psychological resilience, (b) school climate and mathematics achievement, and (c) mathematical bullying and mathematics achievement.

The existing literature provides substantial evidence of bivariate (pairwise) associations among school climate, general bullying, and academic achievement. However, negative interpersonal experiences specific to mathematics lessons have been examined in relatively few studies. The association between mathematical bullying and psychological resilience, and the role of psychological resilience in the pathway to mathematics achievement, remain unclear. Moreover, whether gender and grade level alter these structural associations has not been evaluated within a single model. The present study addresses this gap by estimating serial indirect associations linking school climate with mathematics achievement. It examines the serial indirect associations through mathematical bullying and psychological resilience and whether specific associations within the model vary by gender and grade level. Thus, the study evaluates not only direct and indirect associations among the variables but also whether the strengths of these associations differ across student groups. The model developed for this purpose was tested against hypotheses H1–H5b and is shown in Figure 1.

**Figure 1:**
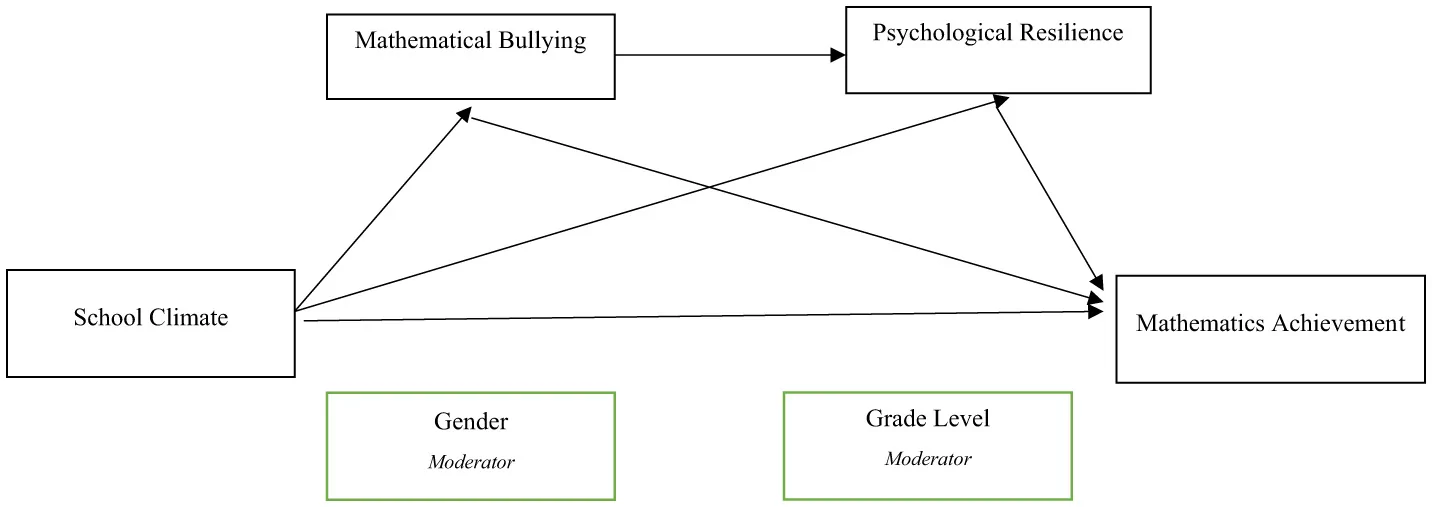
Conceptual model.

## 3 Method

### 3.1 Research design

A cross-sectional, quantitative, and correlational design was used to examine associations among school climate, mathematical bullying, psychological resilience, and mathematics achievement. Cross-sectional designs can provide evidence of associations among variables using data collected within a particular time frame but do not establish temporal ordering or causality (Creswell and Creswell, 2018; Spector, 2019). Accordingly, this study estimated the direct and serial indirect associations between school climate and mathematics achievement through mathematical bullying and psychological resilience, without interpreting these associations as causal.

### 3.2 Participants

The study was conducted in three public middle schools in Kocaeli, Türkiye. Including multiple schools provided contextual diversity and reduced the extent to which the sample was constrained by the climate and student profile of any single school. However, because the schools were selected through convenience sampling, the sample cannot be assumed to be representative of the wider school population. The final sample comprised 708 middle school students: 345 girls (48.7%) and 363 boys (51.3%). By grade level, 147 (20.8%) students were in Grade 6, 204 (28.8%) in Grade 7, and 357 (50.4%) in Grade 8. Participants were recruited via convenience sampling from students who attended schools accessible to the researchers and volunteered. Convenience sampling is based on selecting participants according to accessibility and feasibility (Cohen et al., 2018). Eligibility criteria were enrollment in Grade 6, 7, or 8 at one of the participating schools, and provision of the required student and parental consent. Participation was voluntary, and no personally identifiable information was collected.

### 3.3 Data collection instruments

#### 3.3.1 Brief school climate scale

Students' perceptions of school climate were assessed using the Brief School Climate Scale, developed by White et al. (2014) and adapted into Turkish by Özdogan and Yalçin (2025). The scale comprises nine items and a single factor, with responses provided on a 4-point Likert-type scale ranging from 1 to 4. Higher scores indicate a more positive perception of school climate. Example items include “My school sets clear rules for behavior” and “I know an adult at school that I can talk with if I need help.” In the Turkish adaptation study, the one-factor structure yielded an acceptable fit among middle school students (χ^2^/df = 2.60, CFI = 0.95, TLI = 0.93, RMSEA = 0.07, and SRMR = 0.04), and Cronbach's alpha was 0.83.

#### 3.3.2 Mathematical bullying victimization scale

The mathematical bullying victimization scale (MBVS), developed by Küçükgençay (2025), was used to assess students' exposure to bullying in mathematics contexts. The scale comprises 14 items across four dimensions of bullying: verbal, emotional, physical, and instructional-mathematical. Items are rated on a 5-point Likert-type scale ranging from 1 (strongly disagree) to 5 (strongly agree), with higher scores indicating greater mathematical bullying victimization. An example item is: “Because I am unsuccessful in mathematics, my classmates laugh at me when the teacher asks me to come to the board.” In the development study, the second-order factor structure yielded χ^2^/df = 1.56, CFI = 0.961, TLI = 0.950, RMSEA = 0.059, and SRMR = 0.042; Cronbach's alpha was 0.84. In the present study, the total mathematical bullying score was used because the second-order factor structure supported in the MBVS development study permits the four dimensions to be interpreted as indicators of an overarching mathematical bullying victimization construct (Küçükgençay, 2025). This scoring decision was also aligned with the aim of examining the overall position of mathematical bullying within the proposed serial model, rather than estimating separate pathways for each dimension. Accordingly, the findings represent the shared contribution of the four dimensions within the higher-order construct.

#### 3.3.3 Brief resilience scale

Students' psychological resilience was assessed using the Brief Resilience Scale, developed by Smith et al. (2008) and adapted into Turkish by Dogan (2015). This one-factor scale comprises six items rated on a 5-point Likert-type scale ranging from 1 (strongly disagree) to 5 (strongly agree). Items 2, 4, and 6 are reverse-scored, and higher scores indicate greater psychological resilience. Example items include the following: “I tend to bounce back quickly after hard times” and “I have a hard time making it through stressful events.” The Turkish adaptation study reported CFI = 0.99, RMSEA = 0.05, SRMR = 0.03, and Cronbach's alpha = 0.83.

#### 3.3.4 Mathematics achievement

Students' mathematics achievement was determined from their scores on written examinations, assignments, projects, and other performance-based assessments completed during the spring semester of the 2025–2026 academic year. An overall mathematics achievement score was calculated as the arithmetic mean of each student's assessment scores. In accordance with the assessment and evaluation principles of the Ministry of National Education, scores ranged from 0 (lowest) to 100 (highest). Mathematics achievement was treated as a continuous variable, and no additional standardization was applied. Three mathematics teachers at the participating schools assigned achievement scores to students. Although the teachers followed the same national mathematics curriculum, common learning outcomes, and Ministry assessment principles, each teacher prepared and scored the examinations and performance-based assessments at their school. The resulting scores, therefore, reflect school-based mathematics performance rather than scores on a centrally administered standardized mathematics test.

#### 3.3.5 Control Variables

Previous research suggests that bullying experiences may vary by gender and grade level (Küçükgençay, 2025), while gender may moderate the association between bullying and mathematics achievement (Ren et al., 2025). Gender and grade level were therefore included as control variables in all equations of the regression and serial indirect-association model to account for potential confounding. Gender was dummy-coded (0 = girl, 1 = boy). In the control analyses, grade level was coded as an increasing ordinal variable (0 = Grade 6, 1 = Grade 7, and 2 = Grade 8). In the moderation analyses, grade level was treated as a categorical grouping variable.

### 3.4 Procedure

Data were collected between March 20 and May 20, 2026. The scales were administered face-to-face by the researchers during regular mathematics lessons to ensure that administration conditions were as similar as possible across participants. Before the administration, students were informed of the purpose of the study and how to complete the forms. Mathematics achievement scores were obtained from the relevant mathematics teachers and entered into the dataset. A total of 727 survey forms were collected. Before analysis, the forms were examined for incomplete or erroneous responses and for response patterns that did not meet data-quality criteria. Nineteen forms were excluded, leaving 708 forms that met the required data-quality criteria for the final analyses.

### 3.5 Ethical approval

Students were informed that participation was voluntary, that their responses would remain confidential, and that the data would be used solely for scientific purposes. Informed consent was obtained from the students and their parents, and no identifying information was collected. Ethical approval was obtained from [Kocaeli University Social and Human Sciences Ethics Committee,19.02.2025, E-94094268-020-1014054], and permission to conduct the study was obtained from the relevant institutions.

### 3.6 Data analysis

Confirmatory factor analyses were first conducted to examine the factorial validity of the measures. Descriptive statistics, correlations, and reliability coefficients were then calculated, and regression-based serial indirect association and moderation analyses were conducted. Data were analyzed using IBM SPSS Statistics 29.0, IBM SPSS AMOS 29.0, and PROCESS Macro v4.1. SPSS 29.0 was used to calculate descriptive statistics, Pearson correlation coefficients, and Cronbach's alpha; AMOS 29.0 was used to conduct CFAs. Serial indirect associations and moderation were estimated using Models 6 and 1, respectively, in PROCESS Macro v4.1 (Hayes, 2022). Before analysis, the dataset was examined for missing values and distributional characteristics; no missing values were identified. Means, standard deviations, skewness, and kurtosis were calculated for the study variables, and bivariate associations were evaluated using Pearson correlation coefficients. Skewness and kurtosis values for all variables fell between –2 and +2, supporting the assumption of univariate normality (George and Mallery, 2010). Because students were sampled from three schools, the serial indirect-association model was also re-estimated as a sensitivity analysis, with two dummy-coded school indicators (with School A as the reference group) included as covariates in all three equations. This analysis examined whether the primary model estimates were robust to adjustment for school membership. Given that a very small number of clusters can limit the reliability of multilevel models and cluster-based inference, this adjustment was treated as a fixed-effects sensitivity analysis controlling for between-school mean differences rather than as a full multilevel correction (McNeish and Stapleton, 2016; McNeish and Kelley, 2019). To evaluate robustness to differences in grading practices, mathematics achievement scores were standardized within each school and the model was re-estimated. As a sensitivity analysis, the model was re-estimated with grade level treated categorically, using Grade 6 as the reference group.

Construct validity was examined through separate CFAs for each measurement instrument in AMOS 29.0. Model fit was evaluated collectively using χ^2^/df, CFI, GFI, RMSEA, and SRMR, with commonly used guidelines informing the interpretation of acceptable fit (Kline, 2016). Internal consistency was assessed using Cronbach's alpha, and discriminant validity was evaluated using the heterotrait–monotrait ratio of correlations (HTMT). Harman's single-factor test was applied to the self-report items as a preliminary diagnostic check for potential common method bias. In the unrotated principal components analysis, the first factor accounted for 21.53% of the total variance, indicating that no single factor dominated the variance among the self-report items. Nevertheless, this diagnostic does not rule out common method bias (Podsakoff et al., 2003). A serial indirect-association model estimated the direct and indirect associations between school climate and mathematics achievement, with mathematical bullying and psychological resilience specified sequentially in the indirect-association model. Gender and grade level were included as covariates in all regression equations. The significance of indirect associations was evaluated using bias-corrected 95% bootstrap confidence intervals based on 5,000 resamples. An interval that did not include zero was considered evidence of a statistically significant indirect association. The moderating roles of grade level and gender on specific associations within the model were examined by including interaction terms. Group-based simple-slope analyses were conducted to interpret significant interactions.

## 4 Results

### 4.1 Measurement model

Before testing the hypotheses, the psychometric properties of the three multi-item latent variables—school climate, mathematical bullying, and psychological resilience—were evaluated using CFA. The measurement model fit and reliability results are presented in Table 1.

**Table 1:** Measurement model fit and reliability results.

Scale	*χ^2^*/df	CFI	GFI	RMSEA	SRMR	Factor loadings	Cronbach's α
School climate	1.269	0.974	0.891	0.037	0.067	0.488–0.612	0.693
Mathematical bullying	1.157	0.984	0.894	0.028	0.079	0.409–0.681	0.796
Psychological resilience	1.098	0.998	0.979	0.022	0.048	0.553–0.754	0.784

The CFA results indicated acceptable overall model fit for the three measurement instruments; however, item-level measurement quality was not uniformly strong across all indicators. Standardized factor loadings ranged from 0.488 to 0.612 for school climate, 0.409 to 0.681 for mathematical bullying, and 0.553 to 0.754 for psychological resilience. Some loadings at the lower ends of these ranges were modest. Cronbach's alpha coefficients were 0.693, 0.796, and 0.784, respectively. The internal consistency estimates for mathematical bullying and psychological resilience were acceptable, whereas the school climate coefficient (α = 0.693) was marginal and slightly below 0.70. Accordingly, findings involving school climate and the mathematical bullying score, which included some comparatively low-loading items, should be interpreted cautiously. Discriminant validity was assessed using HTMT. HTMT values were 0.605 for the association between school climate and mathematical bullying; 0.257 for the association between school climate and psychological resilience; and 0.377 for the association between mathematical bullying and psychological resilience. All values were below the 0.85 threshold, supporting the discriminant validity of the constructs (Henseler et al., 2015).

### 4.2 Descriptive Statistics and Bivariate Correlations

Means, standard deviations, and correlations among the study variables are presented in Table 2.

**Table 2:** Descriptive statistics and correlations.

Variable	M	SD	Skewness	Kurtosis	1	2	3	4
1. School climate	3.645	0.657	−0.449	0.513	1			
2. Mathematical bullying	2.036	0.735	1.212	1.369	−0.41^**^	1		
3. Psychological resilience	3.127	0.890	0.093	−0.649	0.26^**^	−0.31^**^	1	
4. Mathematics achievement	61.995	23.932	−0.197	−0.802	0.22^**^	−0.19^**^	0.24^**^	1

^**^p < 0.01.

School climate was negatively correlated with mathematical bullying (*r* = **–**0.41, *p* < 0.001) and positively correlated with both psychological resilience (*r* = 0.26, *p* < 0.001) and mathematics achievement (*r* = 0.22, *p* < 0.001). Mathematical bullying was negatively correlated with psychological resilience (*r* = **–**0.31, *p* < 0.001) and mathematics achievement (*r* = **–**0.19, *p* < 0.001). Psychological resilience was positively correlated with mathematics achievement (*r* = 0.24, *p* < 0.001). These findings provided the correlational basis for estimating the proposed serial indirect-association model.

### 4.3 Direct effect and regression analysis

Multiple linear regression analyses were conducted to examine the direct associations specified in the research model. The analyses assessed school climate as a statistical predictor of mathematical bullying and mathematics achievement; mathematical bullying as a statistical predictor of psychological resilience and mathematics achievement; and psychological resilience as a statistical predictor of mathematics achievement. The results are presented in Table 3.

**Table 3:** Multiple linear regression results.

Outcome variable	Predictor	*R^2^*	B	SE	*t*	*p*	BOOTSTRAP 95% CI	
							Lower	Upper
Mathematical bullying	School climate	0.171	−0.458	0.067	−6.83	< 0.001	−0.590	−0.326
	Gender		0.032	0.088	0.36	0.717	−0.141	0.205
	Grade level		0.018	0.056	0.32	0.746	−0.092	0.128
Psychological resilience	School climate	0.206	0.213	0.087	2.44	0.016	0.041	0.385
	Mathematical bullying		−0.300	0.078	−3.85	< 0.001	−0.453	−0.146
	Gender		0.526	0.104	5.04	< 0.001	0.320	0.732
	Grade level		−0.073	0.066	−1.10	0.273	−0.204	0.058
Mathematics achievement	School climate	0.109	4.890	2.523	1.94	0.054	−0.080	9.860
	Mathematical bullying		−2.563	2.293	−1.12	0.265	−7.081	1.955
	Psychological resilience		4.226	1.877	2.25	0.025	0.528	7.924
	Gender		2.795	3.138	0.89	0.374	−3.388	8.978
	Grade level		−4.052	1.897	−2.14	0.034	−7.790	−0.313

School climate was a statistically significant negative predictor of mathematical bullying (*B* = **–**0.458, *p* < 0.001); neither gender (*B* = 0.032, *p* = 0.717) nor grade level (*B* = 0.018, *p* = 0.746) was a significant predictor. Psychological resilience was positively predicted by school climate (*B* = 0.213, *p* = 0.016) and negatively predicted by mathematical bullying (*B* = **–**0.300, *p* < 0.001). Gender was a statistically significant predictor of psychological resilience (*B* = 0.526, *p* < 0.001), whereas grade level was not (*B* = **–**0.073, *p* = 0.273). In the mathematics achievement model, psychological resilience was a significant positive predictor (*B* = 4.226, *p* = 0.025), whereas grade level was a significant negative predictor (*B* = **–**4.052, *p* = 0.034). The direct associations of school climate (*B* = 4.890, *p* = 0.054), mathematical bullying (*B* = **–**2.563, *p* = 0.265), and gender (*B* = 2.795, *p* = 0.374) with mathematics achievement were not significant. The models accounted for 17.1%, 20.6%, and 10.9% of the variance in mathematical bullying, psychological resilience, and mathematics achievement, respectively.

### 4.4 Serial indirect-association analysis

A serial indirect-association analysis was conducted to examine the separate and sequential indirect associations between school climate and mathematics achievement through mathematical bullying and psychological resilience. The results are presented in Table 4, and the tested model is shown in Figure 2.

**Table 4:** Bias-corrected bootstrap results for direct and indirect effects.

Model paths	Unstandardized coefficient (SE)	Standardized coefficient (SE)	*p*	BOOTSTRAP 95% CI		Proportion (%)
				Lower	Upper	
SC → MB → MA	1.175 (1.137)	0.032 (0.031)	0.281	−0.026	0.098	15.6
SC → PR → MA	0.899 (0.590)	0.025 (0.016)	0.039	0.003	0.066	11.9
SC → MB → PR → MA	0.581 (0.337)	0.016 (0.009)	0.024	0.003	0.040	7.7
Total indirect effect	2.655 (1.259)	0.073 (0.034)	0.024	0.009	0.142	35.2
Direct effects	4.890 (2.839)	0.134 (0.077)	0.084	−0.012	0.289	64.8
Total effects	7.545 (2.411)	0.207 (0.064)	< 0.001	0.082	0.332	100.0

SC, school climate; MB, mathematical bullying; PR, psychological resilience; MA, mathematics achievement. The confidence intervals (95% CIs) refer to standardized coefficients (β) and were calculated using bias-corrected bootstrapping with 5,000 resamples. Standard errors and *p*-values for the direct and total effects in this table are bootstrap-based and therefore differ slightly from the OLS-based values reported in Table 3 for the same coefficient

**Figure 2:**
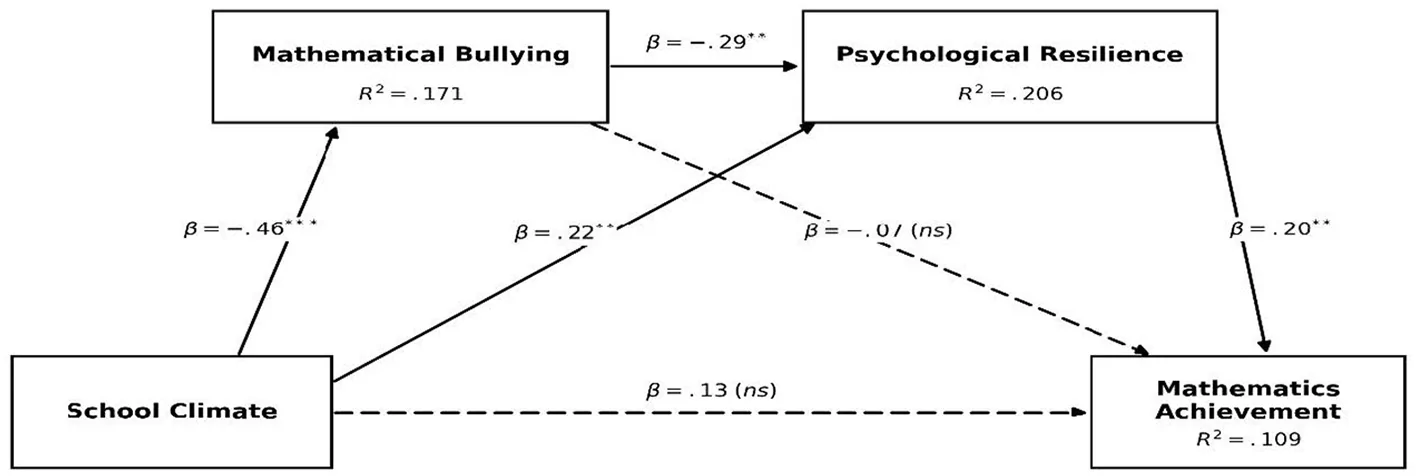
Serial indirect-association model of the association between school climate and mathematics achievement through mathematical bullying and psychological resilience.

The total association between school climate and mathematics achievement was positive and significant [β = 0.207, *p* < 0.001, 95% CI (0.082, 0.332)], supporting H1. The total indirect association was also significant [β = 0.073, *p* = 0.024, 95% CI (0.009, 0.142)] and accounted for 35.2% of the total association. The indirect association through mathematical bullying alone was not significant [β = 0.032, *p* = 0.281, 95% CI (**–**0.026, 0.098)]; therefore, H2 was not supported. By contrast, the indirect association through psychological resilience was significant [β = 0.025, *p* = 0.039, 95% CI (0.003, 0.066)] and accounted for 11.9% of the total association, supporting H3. The serial indirect association through mathematical bullying and psychological resilience was also significant [β = 0.016, *p* = 0.024, 95% CI (0.003, 0.040)], accounting for 7.7% of the total association and supporting H4. The direct association was not statistically significant [β = 0.134, *p* = 0.084, 95% CI (**–**0.012, 0.289)]. These findings should be interpreted as statistical indirect associations rather than evidence of temporal or causal mediation.

### 4.5 Moderation analyses

Under hypotheses H5a and H5b, the moderating roles of grade level and gender on specific associations within the research model were examined. Results for grade level and gender are presented in Tables 5, 6, respectively.

**Table 5:** Moderation by grade level.

Outcome variable	Groups	Predictor	Unstandardized coefficient (SE)	Standardized coefficient (SE)	*p*	BOOTSTRAP 95% CI	
						Lower	Upper
Psychological resilience	Grade 6	Mathematical bullying	−1.094 (0.263)	−0.904 (0.218)	< 0.001	−1.333	−0.475
	Grade 7	Mathematical bullying	−0.333 (0.129)	−0.275 (0.106)	0.010	−0.485	−0.066
	Grade 8	Mathematical bullying	−0.196 (0.094)	−0.162 (0.078)	0.038	−0.315	−0.009
Mathematics achievement	Grade 6	School climate	−1.001 (5.147)	−0.028 (0.141)	0.846	−0.306	0.251
	Grade 7	School climate	19.716 (4.557)	0.542 (0.125)	< 0.001	0.295	0.789
	Grade 8	School climate	−1.471 (3.092)	−0.040 (0.085)	0.635	−0.208	0.127

Bootstrap 95% CIs for standardized coefficients (β) were calculated using 5,000 resamples.

**Table 6:** Moderation by gender.

Outcome variable	Groups	Predictor	Unstandardized coefficient (SE)	Standardized coefficient (SE)	*p*	BOOTSTRAP 95% CI	
						Lower	Upper
Psychological resilience	Girls	Mathematical bullying	−0.510 (0.122)	−0.421 (0.101)	< 0.001	−0.619	−0.223
	Boys	Mathematical bullying	−0.184 (0.093)	−0.152 (0.077)	0.050	−0.303	0.000
	DIFF (Boys–Girls)	Mathematical bullying	0.326 (0.146)	0.269 (0.121)	0.027	0.031	0.508
Mathematics achievement	Girls	School climate	−0.461 (3.575)	−0.013 (0.098)	0.897	−0.206	0.181
	Boys	School climate	9.065 (3.199)	0.249 (0.088)	0.005	0.076	0.422
	DIFF (Boys–Girls)	School climate	9.526 (4.541)	0.262 (0.125)	0.037	0.016	0.508
Mathematics achievement	Girls	Mathematical bullying	2.983 (3.615)	0.092 (0.111)	0.410	−0.127	0.311
	Boys	Mathematical bullying	−5.381 (2.688)	−0.165 (0.083)	0.046	−0.328	−0.003
	DIFF (Boys–Girls)	Mathematical bullying	−8.364 (4.232)	−0.257 (0.130)	0.049	−0.513	−0.001

Bootstrap 95% CIs refer to standardized coefficients (β), calculated using 5,000 resamples.

Grade level significantly moderated the association between mathematical bullying and psychological resilience (*R*^2^ = 0.036, *F* = 5.44, *p* = 0.005). Although the negative association between mathematical bullying and psychological resilience was significant at every grade level, it was strongest in Grade 6. Grade level also significantly moderated the association between school climate and mathematics achievement (*R*^2^ = 0.059, *F* = 8.50, *p* < 0.001). The positive association between school climate and mathematics achievement was significant only in Grade 7. These findings were consistent with H5a.

Gender significantly moderated several associations. The negative association between mathematical bullying and psychological resilience was stronger among girls (β = 0.269, *p* = 0.027). The positive association between school climate and mathematics achievement was significant only among boys (β = 0.262, *p* = 0.037). The negative association between mathematical bullying and mathematics achievement was significant only among boys (β = **–**0.257, *p* = 0.049). These findings were consistent with H5b but should be interpreted cautiously.

### 4.6 Sensitivity analyses

Three sensitivity analyses were conducted to examine the robustness of the findings to school membership, differences in achievement scoring, and grade-level coding. The serial indirect-association model was re-estimated with (a) two dummy-coded school indicators (School A as the reference group), (b) mathematics achievement scores standardized within school, and (c) grade level treated categorically (Grade 6 as the reference group). The school-indicator coefficients are presented in Table 7A, whereas the indirect-association estimates across the primary and sensitivity specifications are presented in Table 7B.

Table 7Sensitivity analyses for the serial indirect-association model.

**Table:** 

A. School-indicator estimates in the school-adjusted specification (School A = reference).				
Outcome equation	School indicator	β [95% CI]	SE	*p*
Mathematical bullying	School B	−0.016 [−0.092, 0.062]	0.039	0.679
Mathematical bullying	School C	0.000 [−0.081, 0.078]	0.039	0.999
Psychological resilience	School B	0.035 [−0.040, 0.110]	0.039	0.356
Psychological resilience	School C	0.035 [−0.041, 0.109]	0.038	0.369
Mathematics achievement	School B	−0.049 [−0.129, 0.030]	0.041	0.229
Mathematics achievement	School C	−0.047 [−0.126, 0.036]	0.041	0.250

**Table:** 

B. Indirect-association estimates across sensitivity specifications.			
Specification	Indirect via bullying β [95% CI]	Indirect via resilience β [95% CI]	Serial indirect β [95% CI]
Primary model	0.032 [−0.026, 0.098]	0.025 [0.003, 0.066]	0.016 [0.003, 0.040]
School-adjusted (dummy-coded)	0.031 [−0.003, 0.067]	0.028 [0.012, 0.050]	0.014 [0.006, 0.026]
Within-school-standardized achievement	0.031 [−0.002, 0.069]	0.028 [0.012, 0.049]	0.014 [0.007, 0.026]
Grade level treated categorically	0.030 [−0.003, 0.065]	0.021 [0.008, 0.042]	0.012 [0.005, 0.023]

Confidence intervals in Panels A and B are bias-corrected 95% bootstrap confidence intervals based on 5,000 resamples.

Neither School B nor School C was statistically significant in any of the outcome equations (Table 7A). Across all three sensitivity specifications, the indirect association through psychological resilience and the serial indirect association remained significant, whereas the indirect association through mathematical bullying remained nonsignificant (Table 7B). However, the inclusion of school dummy variables does not fully model within-school dependence and should not be interpreted as a complete multilevel solution. Because each school was represented by one mathematics teacher, school and teacher effects could not be separated.

As summarized in Table 8, H1, H3, and H4 were supported, whereas H2 was not supported. The findings were consistent with H5a and provided tentative support for H5b.

**Table 8:** Hypothesis-testing results.

Hypothesis	Tested pathway	*p*	Result
H1	SC → MA (total association)	< 0.001	Supported
H2	SC → MB → MA	0.281	Not supported
H3	SC → PR → MA	0.039	Supported
H4	SC → MB → PR → MA	0.024	Supported
H5a	Grade moderates: MB → PR	0.005	Consistent
H5a	Grade moderates: SC → MA	< 0.001	Consistent
H5b	Gender moderates: MB → PR	0.027	Tentatively consistent
H5b	Gender moderates: SC → MA	0.037	Tentatively consistent
H5b	Gender moderates: MB → MA	0.049	Tentatively consistent

## 5 Discussion

This study examined the separate and serial indirect roles of mathematical bullying and of psychological resilience in the association between school climate and mathematics achievement, using a serial indirect-association model. Separate moderation analyses also assessed whether specific associations in the model varied by grade level and gender.

### 5.1 School climate and mathematics achievement

Students' positive perceptions of school climate were associated with higher mathematics achievement. This finding is consistent with studies linking school climate to mathematics achievement, either directly or through student-level psychological resources such as academic self-efficacy (Bodovski et al., 2013; Demir et al., 2026; Hoy and Hannum, 1997; Lee et al., 2026; López et al., 2023; Zysberg and Schwabsky, 2021). Studies and research syntheses focusing on general academic achievement have likewise identified similar associations between positive school climate and achievement (Berkowitz et al., 2017; Demirtaş-Zorbaz et al., 2021; Erdem and Kaya, 2024; Shindler et al., 2016).

However, not all studies have reached the same conclusion. Hall et al. (2025) found no significant association between students' perceptions of school climate and their subsequent mathematics achievement. Similarly, Nalçaci and Bektaş (2016) reported no significant associations between students' perceptions of supportive teacher behaviors, achievement orientation, or a safe learning environment and their academic achievement. Bahçetepe and Meşeci Giorgetti (2015) reported mixed findings across school climate dimensions: supportive teacher behavior and achievement orientation were positively associated with academic achievement, whereas safe learning environment and positive peer interaction were not significantly associated with achievement. Wilson Fadiji and Reddy (2021), however, identified a negative direct association between school climate and mathematics achievement and a positive indirect association through students' educational expectations. Meta-analytic estimates ranging from small to moderate further indicate that findings may vary across studies (Demirtaş-Zorbaz et al., 2021; Erdem and Kaya, 2024).

The present finding demonstrates an association between students' perceptions of their schools as supportive, safe, and orderly and their mathematics achievement. Differences across studies may be related to how school climate is measured, the achievement indicator used, the level of analysis, and the educational context. Moreover, assessing school climate with a total score does not identify which school characteristics are more strongly associated with mathematics achievement. The cross-sectional design also precludes determining the direction of the association. Students who perceive the school climate more positively may attain higher mathematics achievement, but students who are more successful in mathematics may also evaluate their school experiences more positively.

### 5.2 The indirect association through mathematical bullying

Students' positive perceptions of school climate were associated with lower levels of mathematical bullying. This finding is consistent with studies linking positive perceptions of teacher and peer support, clear and fair rules, school safety, and adult intervention in bullying with lower levels of bullying and victimization (Acosta et al., 2019; Aldridge et al., 2018; Biernbaum and Lotyczewski, 2015; Cornell et al., 2015; Gage et al., 2014; Låftman et al., 2017; Mucherah et al., 2018; Zhao et al., 2021). However, not all school climate characteristics are associated with bullying in the same way, and the magnitude of the association may vary according to the information source used (Aldridge et al., 2018; Hultin et al., 2021). The present finding, therefore, points particularly to an association between mathematical bullying and the school's relational, safety-related, and regulatory characteristics.

Research on bullying in specific subject contexts also supports this finding. Particularly in physical education, teaching style, peer support, adults' responses to bullying, and the visibility of students' performance have been associated with bullying and victimization (Borowiec et al., 2022; Jiménez-Barbero et al., 2020; Montero-Carretero and Cervelló, 2020; O'Connor and Graber, 2014; Ugraş et al., 2025; Wei and Graber, 2023). During mathematics lessons, correct answers, calculation speed, and achievement levels can also be compared visually among students. Differences in mathematical competence may therefore become part of interpersonal power relations. Mathematical bullying encompasses ridicule and disparagement based on mathematics performance, avoidance of classroom participation for fear of humiliation, interference with materials, and negative behaviors perceived to originate from the teacher (Küçükgençay and Peker, 2024; Küçükgençay, 2025). The present study extends the established association between school climate and general bullying to these mathematics-specific experiences.

Although mathematical bullying and mathematics achievement were negatively correlated, neither the direct effect nor the indirect effect via mathematical bullying alone was significant after including the other variables in the model. This result differs from studies linking bullying victimization with lower mathematics achievement (Carrasco et al., 2022; Delprato et al., 2017; Konishi et al., 2010; Ren et al., 2025). Nevertheless, the generally small association between bullying and academic achievement, and its attenuation after accounting for school safety, belonging, classroom engagement, or teacher–student relationships, are consistent with the present finding (Carrasco et al., 2022; Liu et al., 2022; Nakamoto and Schwartz, 2010; Ren et al., 2025).

This result does not indicate that mathematical bullying is irrelevant to achievement. The experiences encompassed by mathematical bullying may not be associated with achievement to the same extent, and these associations may vary according to psychological processes or student characteristics. Indeed, the serial indirect path, in which mathematical bullying operates through psychological resilience, and the negative bullying–achievement association identified among boys point to conditional associations that were not evident in the overall sample. Given the cross-sectional design, the possibility that low mathematics achievement increases students' vulnerability to mathematical bullying cannot be excluded. The association between mathematical bullying and achievement should therefore be considered in conjunction with school context, psychological resilience, and student characteristics, rather than as a direct and uniform pattern across all students.

### 5.3 The indirect association through psychological resilience

The indirect path from school climate to mathematics achievement through psychological resilience was significant. A more positive perception of school climate was associated with greater psychological resilience, which in turn, was associated with higher mathematics achievement. This finding indicates that the association between school climate and mathematics achievement is also linked to students' capacity to recover from difficulties.

The positive association between school climate and psychological resilience is consistent with previous research. Teacher support, positive peer relationships, school connectedness, clear rules, and a safe school environment have been associated with greater psychological or academic resilience (Aldridge et al., 2016, 2020; Alva, 1991; Gonzalez and Padilla, 1997; Luo et al., 2023; Sahib et al., 2025; Yan and Li, 2025). OECD findings further indicate that academically resilient students from disadvantaged backgrounds tend to report greater self-confidence, motivation, and more positive approaches to learning (OECD, 2011). More specifically, Gonzalez and Padilla (1997) identified school belonging as a significant predictor of academic resilience among Mexican American high school students. Caring adults, clear boundaries, high expectations, and opportunities for meaningful participation have been emphasized as features of school environments that support student resilience (Benard, 2004; Henderson and Milstein, 2003). Morrison and Allen (2007) similarly highlighted the protective possibilities of classroom, peer, schoolwide, and family–school contexts. More recent findings link teacher–student relationships, school climate, and school resources with academic resilience (Bauer and El-Sayed, 2025; Yang Hansen et al., 2025). Although some of these studies focused on academic rather than general psychological resilience, they provide evidence of an association between environmental support and students' capacity to cope with difficulties.

The positive association between psychological resilience and mathematics achievement is consistent with previous research linking resilience to mathematics or general academic achievement (Demir, 2023; Erduran Tekin, 2024; Nabilah et al., 2024). However, García-Martínez et al. (2021, 2022) found no significant direct association between resilience and academic achievement; instead, they identified indirect associations through academic engagement and self-concept, respectively. Research on academic resilience has identified self-efficacy, control, planning, low anxiety, and persistence as important correlates of resilience (Martin and Marsh, 2006). Mathematics-specific studies have additionally linked resilience with problem solving and mathematics learning outcomes (Attami et al., 2020; Awofala, 2021). However, Turgut Atak and Meriç (2022) found no significant association between psychological resilience and general academic achievement among nursing students. This variation may reflect whether resilience is measured as a general, academic, or mathematics-specific construct, and which achievement indicators are used.

In the present study, resilience was assessed not as a mathematics-specific characteristic but as students' general tendency to recover from difficult experiences. Nevertheless, the significant indirect path is consistent with evidence that resilience mediates the association between school climate and mathematics achievement (Demir et al., 2026). Longitudinal evidence further suggests that a positive school climate may buffer the association between low psychological resilience and subsequent depressive symptoms among adolescents (Zeng et al., 2025). This pattern is consistent with social-ecological approaches to resilience that consider individual psychological resources in relation to environmental conditions (Masten, 2014; Ungar, 2012). However, the cross-sectional indirect-association model does not demonstrate a temporal or causal process. The findings establish associations among school climate, psychological resilience, and mathematics achievement.

### 5.4 The serial indirect association through mathematical bullying and psychological resilience

The serial indirect path from school climate to mathematics achievement through mathematical bullying and psychological resilience was significant. A more positive school climate was associated with lower mathematical bullying; lower mathematical bullying was associated with greater psychological resilience; and greater psychological resilience was associated with higher mathematics achievement. Although the indirect path through mathematical bullying alone was not significant, the serial path was supported when psychological resilience was included in the model, indicating the importance of students' capacity to recover in the relationship between mathematical bullying and achievement.

This result is consistent with research showing that the association between bullying and academic achievement may emerge through psychological and school-related processes. Previous studies have identified school belonging and academic engagement (Li et al., 2020), learning flow (Ren et al., 2025), and psychological difficulties (Murphy et al., 2022) as mediators of the association between bullying and achievement. Findings that bullying victimization is associated with lower psychological resilience also support the mathematical bullying–psychological resilience link in the serial path (Aldridge et al., 2020; Fang et al., 2022; Gkatsa and Rachioti, 2026; Ran et al., 2020). The present study integrates these associations into a single model by including mathematics-specific bullying, general psychological resilience, and mathematics achievement.

The findings integrate Bronfenbrenner (1979) ecological approach and Masten's (2014) psychological resilience framework within a single explanation. School climate represents the social context directly experienced by students; mathematical bullying represents a subject-specific risk experience within that context; and psychological resilience represents students' capacity to recover from difficulties. From this perspective, the serial path indicates that the association between school context and mathematics achievement should be considered together with subject-specific social experiences and students' psychological resources.

Nevertheless, the cross-sectional serial indirect-association model does not establish temporal ordering or causality. Students with lower psychological resilience may perceive negative behaviors more intensely, while students experiencing low achievement may evaluate their social experiences in mathematics lessons and their school climate more negatively. Previous studies also suggest that indirect pathways may vary by gender and educational context (Li et al., 2020; Ren et al., 2025). The identified pathway should, therefore, be regarded not as an invariant mechanism for all students, but as a theoretically coherent sequence of associations requiring examination in longitudinal and multiwave research.

### 5.5 The moderating roles of grade level and gender

Grade level moderated the associations between mathematical bullying and psychological resilience and between school climate and mathematics achievement. The negative association between mathematical bullying and psychological resilience was significant at every grade level but was stronger in Grade 6. The positive association between school climate and mathematics achievement was significant only in Grade 7.

Küçükgençay and Peker (2024) found that total mathematical bullying scores did not differ by grade level. Küçükgençay (2025) likewise found no significant difference in total scores but reported higher instructional bullying in Grade 8 than in Grade 7. Whereas those studies compared students' reported levels of mathematical bullying, the present study examined whether the strength of the association between mathematical bullying and psychological resilience varied by grade level. Similar total levels of bullying across grades, therefore, do not imply that bullying is associated with psychological resilience equally across grade levels.

The stronger association between mathematical bullying and psychological resilience in Grade 6 may be related to greater sensitivity among students to peer evaluations of their mathematical competence. However, because cross-sectional data do not demonstrate developmental change, this explanation should be treated with caution. The finding that school climate was associated with mathematics achievement only in Grade 7 indicates that it was more strongly linked with achievement at that grade level. Both conditional findings require replication in other samples.

Gender moderated three associations in the model. The negative association between mathematical bullying and psychological resilience was stronger among girls, whereas only among boys the positive association between school climate and mathematics achievement and the negative association between mathematical bullying and mathematics achievement were significant. This pattern indicates that gender did not moderate all associations in the same direction. The stronger mathematical bullying–psychological resilience association among girls may be considered alongside studies reporting higher total and emotional mathematical bullying scores among girls (Küçükgençay and Peker, 2024; Küçükgençay, 2025). However, previous studies examined exposure levels, whereas the present study assessed the strength of the association between two variables. Alongside studies reporting stronger psychological consequences of bullying among girls, other studies have found no gender differences in particular associations involving victimization, psychological resilience, and psychological wellbeing (Bakker et al., 2010; Lin et al., 2022; Wang and Zhang, 2022). Thus, this result does not indicate that girls generally have lower psychological resilience, but rather that the association examined was stronger among girls in this sample.

The finding that the association between school climate and mathematics achievement was significant only among boys does not mean that boys perceived the school climate more positively. Gender has been reported to play different roles in school-climate-related processes. For example, the direct association between competitive school climate and bullying was more pronounced among girls, whereas the indirect association through belonging was more pronounced among boys (Huang et al., 2024). The finding that the mathematical bullying–mathematics achievement association was significant only among boys is consistent with Gimenez et al. (2024), whereas Ren et al. (2025) reported a stronger association among girls. These differences indicate that the level of exposure to bullying should be distinguished from its associated academic and psychological correlates. They also suggest that the moderating role of gender may vary according to the type of bullying and the educational context.

### 5.6 Theoretical and practical implications

#### 5.6.1 Theoretical implications

The principal theoretical contribution of this study is its explanation of the association between school climate and mathematics achievement, not as a direct, single-stage connection but as one that occurs in conjunction with mathematical bullying and psychological resilience. Previous research has largely linked school climate separately with academic achievement, bullying, and psychological resilience, whereas studies of bullying and mathematics achievement have emphasized general bullying measures and processes such as belonging, engagement, and teacher–student relationships (Aldridge et al., 2020; Berkowitz et al., 2017; Carrasco et al., 2022; Ren et al., 2025). The present model brings contextual, social, and psychological associations together in relation to mathematics achievement.

The distinction among the specific indirect pathways is central to this contribution. Although the path through mathematical bullying alone was not significant, the path through psychological resilience alone and the serial path through mathematical bullying and psychological resilience were significant. This result does not diminish the importance of mathematical bullying; rather, it indicates that its association with achievement cannot be explained independently of students' capacity for psychological recovery. The direct association between mathematical bullying and achievement was not significant in the overall sample but significant among boys, whereas its association with psychological resilience was stronger among girls. These findings further demonstrate the need to consider its academic and psychological correlates with caution.

The study also defines the concept of bullying in the context of mathematics learning. Mathematical bullying involves the transformation of differences in mathematical knowledge and achievement into interpersonal abuse of power; it includes disparagement based on performance, avoidance of classroom participation, interference with materials, and negative behaviors perceived to originate from the teacher (Küçükgençay and Peker, 2024; Küçükgençay, 2025). Research in physical education has shown that bullying is associated with subject-specific conditions such as peer support, weight status, and the visibility of performance (Borowiec et al., 2022; O'Connor and Graber, 2014). From the perspectives of Bronfenbrenner (1979) ecological approach and Masten's (2014) resilience framework, the model integrates school context, subject-specific social risk, and individual capacity for recovery within a conditional network of associations.

#### 5.6.2 Practical implications

The findings indicate that efforts to support mathematics achievement should attend not only to instructional practices but also to the provision of safe, orderly, and supportive school environments. The associations of mathematical bullying with school climate and psychological resilience highlight the need for general anti-bullying efforts to consider experiences specific to mathematics lessons. Mathematics teachers and school counseling services can monitor for disparagement based on achievement, ridicule of incorrect answers, avoidance of classroom participation, and interference with students' materials. Classroom environments in which errors are treated as part of learning and different solution strategies are welcomed may support safer participation.

The indirect association through psychological resilience suggests that bullying prevention and support for students' psychological resources should be addressed together. However, responsibility for coping with bullying should not be placed on students; school rules, teacher practices, and peer relationships should remain the primary targets of intervention. Findings concerning grade level and gender should be used not to assign fixed characteristics to particular groups, but to avoid overlooking risks that may emerge among different students. Given the cross-sectional design, these recommendations should be treated as areas for consideration in practice rather than as validated intervention outcomes.

### 5.7 Limitations and directions for future research

This study has several limitations. First, the cross-sectional design does not permit interpreting the ordering of variables specified in the serial indirect-association model as a temporal or causal process. In addition, conducting the study in only three public middle schools selected through convenience sampling limits the generalizability of the findings to other school types and educational contexts. Because students were clustered within schools, the independence of observations could not be fully assumed. Future research may use longitudinal and multilevel designs with larger samples of schools. Although the principal findings were retained in the sensitivity analysis that included school indicators as covariates, the inclusion of only three schools precluded reliable estimation of school-level variation and separation of school and teacher effects.

Mathematics achievement scores were assigned by a different teacher at each school. Although a common curriculum and national assessment principles were implemented, differences in examination difficulty and scoring practices could not be fully controlled. Because each school was represented by a different teacher, school and teacher effects could not be disentangled. Findings concerning mathematics achievement should therefore be interpreted as school-based achievement indicators. Future studies could use a common achievement test or control for score differences across teachers and schools through equating procedures.

School climate, mathematical bullying, and psychological resilience were assessed through reports from the same students; consequently, the identified associations may have been affected by common-source variance. Student reports could be supplemented with teacher assessments, peer assessments, and classroom observations. Mathematical bullying is also a relatively new construct and has been assessed using a single instrument. The measurement properties of the scale should be re-examined across different student groups and educational contexts. The use of total scores also precluded the examination of specific associations between different aspects of school climate and the dimensions of mathematical bullying. The findings should therefore be interpreted as overall associations involving the higher-order mathematical bullying victimization construct, rather than as evidence that the verbal, emotional, physical, and instructional-mathematical dimensions are interchangeable. Future research should examine dimension-specific associations separately. Finally, grade level and gender were tested separately, but only on specific pathways. Therefore, the moderation findings should not be interpreted as indicating that the entire serial indirect effect differed across groups; they should be re-examined in independent samples.

## 6 Conclusion

By examining mathematical bullying, school climate, psychological resilience, and mathematics achievement within the same model, this study demonstrated how social experiences specific to mathematics lessons are connected with broader school and student characteristics. A more positive school climate was associated with lower levels of mathematical bullying, which, in turn, was associated with higher psychological resilience. The serial indirect path through mathematical bullying and psychological resilience indicates that the role of mathematical bullying in the association between school climate and mathematics achievement cannot be evaluated independently of students' psychological resources. Variation in some associations by grade level and gender further indicates that these connections are not uniform across all students. Mathematical bullying is thus positioned not merely as individual victimization but as a phenomenon specific to the mathematics learning environment and associated with school climate and psychological resources.

## Data Availability

The raw data supporting the conclusions of this article will be made available by the authors, without undue reservation.
